# Analyzing the Magnitude of Interlimb Asymmetries in Young Female Soccer Players: A Preliminary Study

**DOI:** 10.3390/ijerph18020475

**Published:** 2021-01-08

**Authors:** Javier Raya-González, Filipe Manuel Clemente, Daniel Castillo

**Affiliations:** 1 Faculty of Health Sciences, Universidad Isabel I, 09001 Burgos, Spain; rayagonzalezjavier@gmail.com (J.R.-G.); danicasti5@gmail.com (D.C.); 2 Sports Sciences Department, Instituto Politécnico de Viana do Castelo, Escola Superior de Desporto e Lazer, Melgaço, 4900-347 Viana do Castelo, Portugal

**Keywords:** fitness testing, interlimb differences, risk factor, football

## Abstract

Although asymmetries in lower limbs have been linked with players’ performance in male soccer players, literature that has been published addressing female soccer is scarce. Thus, the aim of this study was twofold: (i) describe the asymmetries of women soccer players during jumping, change-of-direction and range-of-motion tests; and (ii) test possible relationships between asymmetries and injury risk in female soccer players. Sixteen female players (15.5 ± 1.5 years) performed a battery of fitness tests (i.e., jump ability, change-of-direction ability and passive range-of-motion) and muscle mass analysis via dual-energy X-ray absorptiometry, through which the specific asymmetry index and the related injury risk were calculated. Significant (*p* < 0.05) lower asymmetries in the change-of-direction test were observed in comparison to those observed in jumping and range-of-motion tests; significant (*p* < 0.05) lower asymmetries in muscle mass were also reported compared to those found in the change-of-direction and countermovement jump tests. Additionally, increased injury risk for countermovement jump and hip flexion with extended knee range-of-motion (relating to asymmetry values) and for ankle flexion with flexed knee range-of-motion in both legs (relating to reference range-of-motion values), as well as increased individual injury risk values, were observed across all tests. These findings suggest the necessity to implement individual approaches for asymmetry and injury risk analyses.

## 1. Introduction

Being asymmetric during movement is common since the human body is also asymmetric from an anatomic and neurological point-of-view [1]. The predominance of one limb over the other is usual in many sports, and for that reason, asymmetries between limbs are commonly seen in different sports [2,3,4]. Actually, in some cases, being asymmetric seems to play an important role for performance, in particular, in elite sports [5]. Therefore, a given level of asymmetry may be considered functional. However, there is a natural threshold in which the asymmetry may lead to an increase in risk for each player [6]. Possibly, this threshold is individual for each player and it is important to consider meaningful variations of this in intra-individual analysis [7].

The presence of asymmetry can be screened in different dimensions of analysis. Some of the possibilities can be related to identifying asymmetry in passive range-of-motion (ROM) tests [8] such as hip flexion with extended knee (HFEK), hip abduction with flexed knee (HAFK), ankle flexion with extended knee (AFEK), and ankle flexion with flexed knee (AFFK). Asymmetries can be also found in highly demanding neuromuscular tests that are traditionally used as indicators of fitness status in some sports [9]. In the specific case of soccer, among others, typical neuromuscular tests are related to jumping performance (e.g., countermovement jump (CMJ), horizontal jump (HOP) or lateral jump (LAT) [10,11] or change-of-direction (COD) [12]). Due to the differences observed in the magnitude of the asymmetry of these tests, some authors have stated the necessity to apply a fitness test battery for soccer players in order to provide a holistic picture of the asymmetries in this population [13,14].

Beyond the fact that asymmetries do not meaningfully affect performance in soccer [9,15,16], there are still some concerns about possible relationships between asymmetry levels and the occurrence of injuries. A cohort study tested possible relationships between asymmetry levels (assessed by functional movement screen) and injury likelihood, although no evidence for such relationships was found [17]. However, when using a balance test for the screening of asymmetries, a possible relationship with increases in non-contact injuries was found [18]. Using isokinetic tests of the knee, a study conducted in soccer players failed to predict hamstring injuries [19]. Interestingly, despite continuous trials to find an association between asymmetries in injury risks in soccer players, it seems that the evidence is not line with expectations. However, the above-mentioned studies did not used fitness tests to screen asymmetries. Additionally, the majority of them are conducted with male athletes, with only one study focused on female soccer players [14]. For that reason, it is important to explore possible relationships between asymmetry levels screened in fitness tests with injury occurrence in women soccer players in order to get specific information to prescribe specific preventive programs.

On this basis, the purpose of this study was twofold: (i) describe the asymmetries of women soccer players during jumping, COD and ROM tests; and (ii) test possible relationships between asymmetries and injury risk in female soccer players. Based on previous studies [13,14], we hypothesize that CMJ presents the highest asymmetry magnitude, with poor relation between different asymmetry scores. Additionally, greater individual differences relating to injury risk are expected.

## 2. Materials and Methods

### 2.1. Participants

A convenience sample of sixteen U-17 female soccer players (age: 15.5 ± 1.5 years, height: 158.9 ± 6.9 cm, body mass: 54.62 ± 7.76 kg, body mass index (BMI): 21.7 ± 2.3) belonged to the same team participated in this study. All players trained at least three times a week and were involved in an official match every weekend and presented 4 ± 2 years of soccer experience. Players only participated in training and matches related to their team. Prior to participating in the study, each player completed a comprehensive medical and injury history questionnaire. Goalkeepers were excluded from the statistical analysis due to their specific role. Inclusion criteria required that players complete all the tests, whereas those who were injured during the two months prior to the testing sessions were not included in the final analysis. All the participants and their respective parents or legal guardians were informed of the procedures, methods, benefits, and possible risks of participating in the study before giving their written consent (parents). Additionally, the investigation was carried out under the consent of the soccer club and was conducted according to the Declaration of Helsinki and approved by ethics committee of University Isabel I (Code: FUi1-PI002).

### 2.2. Procedures

A descriptive and correlational design was used to measure interlimb asymmetries from a test battery in young female soccer players, determining the association between asymmetry values and analyzing the group and individual injury related to each test. Two consecutive weeks were selected to perform the testing sessions, with the first one dedicated to familiarizing players with the test battery in order to avoid any learning effects [20]. During the second week, the assessment of the participants’ physical fitness was performed in two different testing sessions (i.e., 48 h of separation between them). In the first session, CMJ, standing broad jump (SBJ), LAT and change of direction ability (CODA) (i.e., 505-COD) tests were carried out in the regular soccer field (i.e., artificial grass), while in the second one, female players completed the lower limb ROM and muscle mass (i.e., dual-energy X-ray absorptiometry (DXA)) assessments in the sport performance lab. Both testing sessions were supervised by the same strength and conditioning coaches, who verbally discussed the study with each participant to ensure both parties were satisfied with requirements before data collection [13]. To estimate the injury risk, specific reference values were selected for each test [21,22,23,24,25]. Prior to the testing sessions, a standardized warm-up consisting in 5 min of slow jogging, followed by 8 min of strolling locomotion and finishing with 5 min of jumps and progressive sprints and accelerations or ballistic dynamic movements, depending on the testing session (i.e., on-field or lab), was carried out. Testing sessions were performed in the afternoon (6–8 p.m., 17–22 °C, 60–70% humidity) during the in-season period (i.e., October).

#### 2.2.1. Jumping Performance Tests

Jump testing comprised CMJ, SBJ and LAT jump tests. Two maximal trials with each leg (i.e., dominant and non-dominant) were performed for each test, separated by 45 s of passive recovery [13]. During the CMJ, all participants were instructed to place their hands on their hips which was followed by a vertical jump at maximal effort and landing in a vertical position, with their knees being flexed after landing [26]. For SBJ and LAT jumps, participants started from a standing position, swinging their arms and bending their knees to provide maximal forward frontal and lateral drive, respectively. CMJ trials were performed on a platform with infrared rays (Optojump Next; Microgate^®^, Bolzano, Italy), while SBJ and LAT jumps were measured through a metric tape, with the jump-length determined from the take-off line to the nearest point of landing contact (i.e., back of the heels) [27]. The best of the performances of each test (i.e., the highest height for CMJ and the longest distance for SBJ and LAT) was selected for the subsequent statistical analysis.

#### 2.2.2. Change of Direction Ability (CODA)

After the jumping assessment, participants performed 4 trials of the 505-COD test (i.e., 2 trials turning to the dominant leg and another two turning to the non-dominant leg) in order to evaluate their CODA. This test consisted of players, after running 10 m without the ball, sprinting forward to a line 5 m ahead and pivoting 180° before returning to the start position [28]. A photocell (Microgate, Bolzano, Italy) located over the start/finish line was used to record the time. The recovery time was stablished in 1.5 min between each sprint, and the fastest time of each test was chosen for the final analysis.

#### 2.2.3. Passive Range of Motion (ROM) Tests

A battery of passive ROM tests was performed following the methodology previously described [29]. The selected tests were HFEK: hip flexion with extended knee; HAFK: hip abduction with flexed knee; AFEK: ankle flexion with extended knee; AFFK: ankle flexion with flexed knee, which have previously demonstrated high reliability for team sport athletes [8]. Each passive ROM test was performed two times (30 s between them) for each leg using a valid laser-guided digital goniometer (HALO medical devices, Australia) [30]. All tests were carried out under stable environmental conditions by the same two physical therapists, whose role was always the same, and the goniometer was recalibrated before each trial. To determine the endpoint for each test, one or both of the following criteria were accomplished: (a) palpable onset of pelvic rotation, and/or (b) the participant feeling a strong but tolerable stretch, slightly before the occurrence of pain. Mean score values from each test and limb were calculated and used for further analysis.

#### 2.2.4. Muscle Mass via Dual-Energy X-ray Absorptiometry (DXA)

The DXA scanner GE/Lunar Prodigy densitometer (GE Healthcare, Madison, WI, USA) with the QDR-Explorer software (pediatric version of the software QDR-Explorer, Hologic Corp. Software version 12.4, Bedford, MA, USA) was used to determine the lower limbs’ muscle mass (in kg), following the DXA best practice guidelines described previously [31]. Calibration tests with a spine phantom were performed daily before taking any measurements. The participants were in a supine position with their hands level with their hips and their feet slightly apart. They were measured with minimum clothes and without jewelry, and velcro straps were used to hold their feet in place with their toes pointed upwards. Additionally, participants had not eaten anything at least 2-h before testing and had not trained. All DXA scans were conducted and analyzed by at least one of the two certified and trained DXA technicians, who positioned the participants, performed the scan, and executed the analysis in a routine clinical manner following the research facility’s standard operating procedures and according to the manufacturer’s guidelines.

### 2.3. Statistical Analysis

Data are presented as mean ± standard deviations (SD). To confirm the normality of data distribution, the Shapiro–Wilk test was used. Within-session reliability of test measures was calculated using the intraclass correlation coefficient (ICC) with absolute agreement and the coefficient of variation (CV). Interpretation of ICC and the 95% confidence interval were calculated and categorized as excellent (0.90–1.00), good (0.75–0.9), moderate (0.50–0.75), or poor (<0.50) [32], and CV values lower than 10% were considered as acceptable [33]. For each test and participant, the limb showing the better score (i.e., the lower score for 505-COD test and the larger score for the remaining tests) was defined as dominant, whereas the opposite limb was defined as non-dominant [13]. Interlimb asymmetries were calculated using a standard percentage difference equation for all the tests: (score in stronger limb—score in weaker limb)/(score in stronger limb) × 100 [34]. When depicting interlimb differences individually, the use of an “IF function” in Microsoft Excel was added to the end of the formula: *IF (left < right, 1, −1) [35], in order to show the direction of asymmetry (i.e., which leg produced the better score) without altering the magnitude. A one-way repeated-measures analysis of variance (ANOVA) was conducted to determine the between-test differences in asymmetry magnitudes, and Pearson’s *r* correlations were conducted to establish the relationship between the tests’ interlimb asymmetry scores. The following scale of magnitude was used to interpret the correlation coefficients: <0.1, trivial; 0.1–0.3, small; 0.3–0.5, moderate; 0.5–0.7, large; 0.7–0.9, very large; and >0.9, nearly perfect. The significance level was set *p* < 0.05. All statistical tests were performed using the software package IBM SPSS Statistics, version 25.0 (IBM Corp., Armonk, NY, USA).

## 3. Results

Table 1 shows the descriptive data and the within-session reliability data. All tests reported good or excellent reliability and an acceptable CV for all the tests (<10%) was obtained.

Between-test differences in asymmetry magnitudes and the associated injury risks are shown in Table 2. Significantly lower values (*p* < 0.05) were observed in 505-COD test compared to jumping test and ROM values. Additionally, the muscle mass (MM) test reported significantly lower values (*p* < 0.05) compared to 505-COD and CMJ. Asymmetry values of increased injury risk were reported for CMJ and ROM_HFEK.

Pearson’s *r* correlations between interlimb asymmetry scores across tests are shown in Table 3. No significant (*p* > 0.05) relationships were reported between asymmetry scores for any test.

Table 4 shows the reference values and the associated injury risk for each ROM test differentiating between dominant and non-dominant legs. No injury risk for any test and limb was reported relating to reference values except for ROM_AFFK.

**Figure 1:**
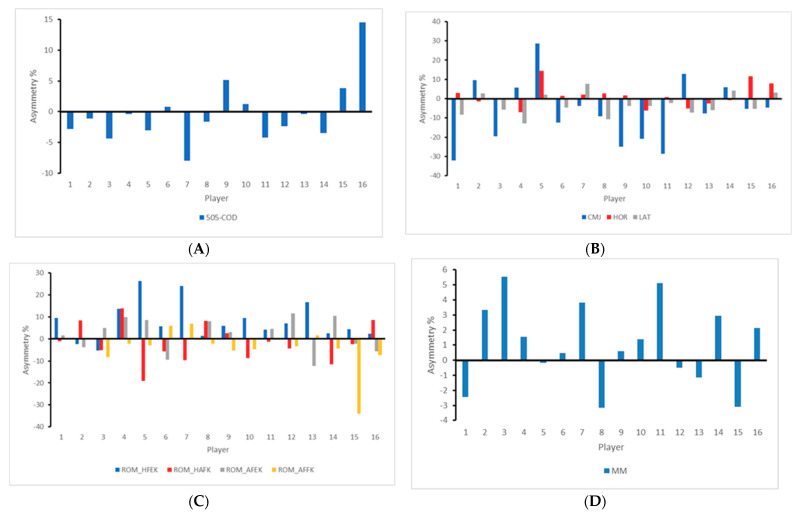
Individual asymmetry data for battery test ((**A**) change of direction test; (**B**) jump test; (**C**) range of motion tests; (**D**) muscle mass). Above the line indicates raw score is greater on the right limb, and below the line indicates raw score is greater on the left limb. 505-COD: 505 change of direction test. CMJ: countermovement jump; SBJ: standing broad jump; LAT: lateral jump. ROM: range of motion; HFEK: hip flexion with extended knee; HAFK: hip abduction with flexed knee; AFEK: ankle flexion with extended knee; AFFK: ankle flexion with flexed knee. MM: muscle mass.

Individual asymmetry values for each player are presented in Figure 1. Individual asymmetry values ranged from 0.39 to 14.51 in 505-COD test, 3.77 to 32.12 in CMJ, 0 to 14.29 in SBJ, 1.96 to 12.86 in LAT, 1.32 to 26.37 in ROM_HFEK, 0 to 19.10 in ROM_HAFK, 0 to 12.20 in ROM_AFEK, 0 to 34.04 in ROM_AFFK, and 0.18 to 5.54 in MM.

**Figure 2:**
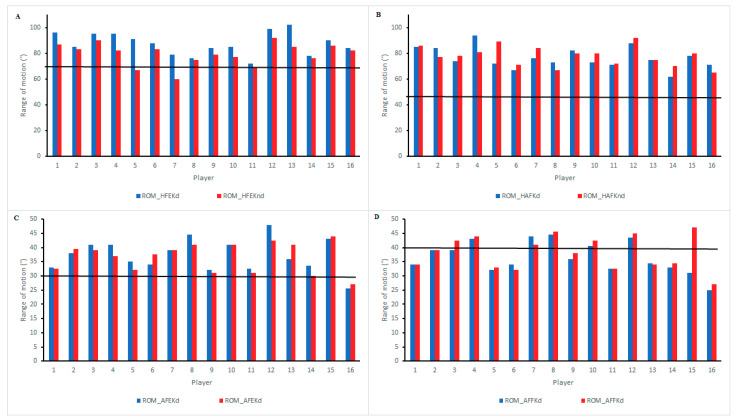
Individual range of movement values ((**A**) flexion with extended knee; (**B**) hip abduction with flexed knee; (**C**) ankle flexion with extended knee; (**D**) ankle flexion with flexed knee). ROM: range of motion; D: dominant; ND: non-dominant; HFEK: hip flexion with extended knee; HAFK: hip abduction with flexed knee; AFEK: ankle flexion with extended knee; AFFK: ankle flexion with flexed knee.

Figure 2 reports individual ROM values for each player and the associated injury risk. In ROM_HFEK, two players presented ROM values related to high injury risk in their non-dominant leg, in ROM_HAFK all players presented ROM values related to high injury risk, in ROM_AFEK one player presented ROM values related to high injury risk in both dominant and non-dominant legs, and in ROM_AFFK eleven players presented ROM values related to high injury risk in their dominant leg and nine in their non-dominant leg.

## 4. Discussion

Since greater values of interlimb asymmetries are associated with higher injury risk in a sport performance context, the aim of this study was to measure interlimb asymmetries from a battery of fitness tests in young female soccer players, determining the association between asymmetry values and analyzing the group and individual injury risk related to each test. The main novelty of this study is that this is the first investigation that performed a comprehensive analysis of COD, multidirectional jumping (i.e., CMJ, SBJ and LAT), lower limb ROM and MM asymmetries in young female soccer players, including an individual analysis and the associated injury risk based on reference values previously identified in the literature [21,22]. The main results of this study show (1) all tests had good–excellent reliability and acceptable consistency; (2) lower asymmetries in the 505-COD test in comparison to jumping and ROM tests, and lower asymmetries in MM compared to 505-COD and CMJ; (3) no significant relationships among asymmetries across different tests; (4) an increase in injury risk for CMJ and ROM_HFEK relating to asymmetry values and for ROM_AFFK in both legs relating to reference ROM values; (5) individual injury risk values were observed across all tests.

Showing high values of reliability (i.e., near to 1) and acceptable consistency (i.e., <10%) is crucial for a fitness test battery in a soccer context in order to assess the players’ physical performance [32]. In our study, all tests presented good–excellent reliability and acceptable consistency, which is in consonance with previous literature in soccer in which similar reliability and consistency values were observed in male [13,35,36] and female [11,37] players. Accordingly, our results in ICC parameters revealed good–excellent conditions suggesting that structured strength and conditioning training could contribute to the good reliability of the data [36]. When asymmetries are considered, previous research has suggested that thresholds of 10% in physical testing and body composition assessment [21], and 8% in lower limbs’ ROM measures [22], might be considered as cutoffs where reductions in performance and heightened injury risk occur. As such, our study revealed the higher magnitudes of asymmetries and consequently, an increased injury risk in the CMJ test. Likewise, the great magnitude of ROM_HFEK asymmetry revealed its relationship with injury risk in hamstrings muscles. Since soccer players are required to have higher levels of flexibility from hip flexors during the backswing phase of the instep kicking action [38], these differences in asymmetries could be explained by this kicking action with the dominant leg. Additionally, our finding provided asymmetry data about MM in dominant and non-dominant lower limbs that could be interesting for further research. Our findings could help strength and conditioning specialists to stablish the appropriate battery of fitness tests in order to detect asymmetries in lower limbs in female soccer players.

Bearing in mind the comparisons in asymmetries resulting from fitness testing, our study found larger magnitudes of asymmetries in jumping tests in comparison with 505-COD test and MM measures. These results coincide with previous studies in soccer that, on one hand, indicated higher asymmetries in jumps rather than in CODA tests [13,39,40] and, on the other hand, reported asymmetries of ~10–15% for vertical [11,15,41], ~4% for horizontal [11,15] and ~5% for lateral [15] jumps. Thus, it seems that monitoring jump asymmetries is relevant in soccer players and concretely measuring jump height asymmetries could help strength and conditioning specialists to develop sport-specific training programs focused on reducing interlimb asymmetries aimed at reduce the injury risk [35]. On the other side, lower limb ROM values showed larger magnitudes of asymmetry than the 505-COD test, showing that ROM assessments could be a better practice in comparison to CODA tests in order to detect asymmetries and, consequently, injury risk. Additionally, it would be interesting for further research to investigate whether other CODA nature tests, with different characteristics in terms of number of CODs, distances or starting speed, are able to detect larger magnitudes of asymmetries in soccer players.

No significant relationships were reported between asymmetry scores for any fitness tests (i.e., CODA, jumping, ROM measures and MM), highlighting the independent nature of the selected tests in young female soccer players (Table 3). Our findings are in consonance with those studies that indicated a lack of relationships between different asymmetry scores in adult and young female soccer players [9,11,14]. Likewise, other authors supported the finding that when comparing asymmetry scores across multiple tests, levels of agreement were typically poor [42]. Whereas the aforementioned studies focused on female soccer have analyzed the asymmetries across selected physical tests, to the best of our knowledge, no previous research has associated interlimb asymmetries based on passive ROM measurements or MM. In this sense, our results also revealed no significant relationships between the different interlimb asymmetry scores based on these measures. So, further research would be convenient to choose those tests or measures that allow strength and conditioning specialists to explain the interlimb asymmetries. Up to date, as previous research has stated, asymmetries are independent of each other, reflecting the necessity to introduce different tests to provide an holistic picture of the asymmetries in female soccer players, as well as to preclude the use of a single test to screen for interlimb asymmetry [13,14]. Therefore, the analysis of asymmetries should be carried outusing multiple tests based on different tasks rather than a single test alone because it does not provide a complete picture of muscular imbalances in female soccer players.

Considering the necessity to provide a more comprehensive injury risk analysis, ROM deficit has been considered as a primary risk factor for some soccer injuries [22]. As such, when reference values are evaluated and related with potential thresholds of injury risk, flexibility training should be prescribed to players with reduced ROM to lower the risk of developing a muscle strain injury [43,44]. In our study, no injury risk for any test and limb were reported in relation to reference values except for ROM_AFFK both in dominant and non-dominant lower limbs. This measure presented lower angles than the reference value proposed by Norris [25] when assessing the soleus muscle. Therefore, it seems clear that it is necessity to prescribe flexibility exercises aimed at improving ROM_AFFK within everyday training routines. In addition, it would be interesting not to neglect the flexibility of those muscles which do not present injury risk according to reference values [23,24,25].

Since soccer players are exposed to greater short-term and high-intensity actions such as accelerations, decelerations, CODs and kicks as well as quick unilateral jumps and sprints during match-play [45], this team sport could be considered as multidirectional in nature as these actions occur unilaterally and are unlikely to be performed in equal amounts using both limbs [46]. So, it seems that asymmetries could be expected in soccer players [42]. Our aforementioned results have showed the interlimb asymmetries scores in a whole group of female soccer players, however, an individualized analysis of asymmetries in each player would be appropriate in order to establish specific preventive programs that could be the key to obtaining positive benefits. In this sense, previous research has postulated the need of prescribing preventive strategies with an adequate degree of compliance because if these strategies are not applied properly, the number of injuries may not be reduced throughout the season [47]. In addition, individual analysis provides information about which asymmetry values favor the left limb (as represented by negative scores) and which favor the right limb (positive asymmetry outcome) for each player, highlighting how a limb may be favored over the other from task to task [13]. This further highlights the individual nature of asymmetries and the requirement for multiple tests [48,49] in order to include effective preventive plans that are individual to each player [11,35,42].

This study is not exempt from limitations, the main one being that only biological age was considered; however, the different maturation stages could influence the obtained results. Given that potential associated alterations in motor control of players [50], future analysis in youth athletes should aim to include this in the analysis. Another limitation was the sample size of sixteen young female players from the same soccer academy who participated in this study; it would be interesting for further studies to involve a greater number of female players from different clubs. Morover, this might compromise the extrapolation of our findings to other populations, especially when considering older or male players. Additionally, equipment used for establishing interlimb asymmetries in 505-COD (e.g., photocells), CMJ (e.g., platform with infrared rays), SBJ and LAT (e.g., metric tape) and ROM measures (e.g., laser-guided digital goniometer) should be taken into account when comparing results with other investigations. Finally, it is possible that the level of the physical conditioning at this stage of competitive season may impact the results, suggesting that future studies choose the best or mean score of fitness testing across the season.

## 5. Conclusions

This study suggests that the magnitude of asymmetries vary across the different fitness tests, ROM values and body composition measures, with CMJ showing the largest asymmetry values in young female soccer players. Consequently, the associated injury risk varies according to the test used suggesting the necessity to establish a fitness battery test. Additionally, no relationships between asymmetry scores were observed, so the application of different tests seems necessary to provide a holistic picture of the asymmetries in female soccer players. Finally, individual asymmetry scores and injury risk values were vastly different from mean values for all metrics, so individual analysis is warranted to implement specific preventive programs for each female soccer player to consider their personal characteristics.

## Figures and Tables

**Table 1:** Descriptive data (mean ± standard deviations (SDs)) and test reliability (95% CI).

Fitness Test	Mean ± SD	ICC (95% CI)	CV (95% CI)
505-CODd (s)	2.54 ± 0.09	0.79 (0.48–0.89)	2.9 (2.2–4.5)
505-CODnd (s)	2.63 ± 0.14	0.72 (0.43–0.87)	4.0 (3.0–6.1)
CMJd (cm)	12.59 ± 2.67	0.96 (0.91–0.98)	3.9 (3.0–6.0)
CMJnd (cm)	10.61 ± 1.76	0.84 (0.66–0.93)	5.5 (4.2–8.5)
SBJd (cm)	139.44 ± 11.10	0.96 (0.91–0.98)	2.0 (1.5–3.1)
SBJnd (cm)	133.81 ± 14.97	0.82 (0.62–0.92)	4.7 (3.5–7.2)
LATd (cm)	140.13 ± 15.44	0.97 (0.93–0.99)	2.2 (1.6–3.3)
LATnd (cm)	132.06 ± 13.95	0.94 (0.86–0.97)	3.0 (2.2–4.5)
ROM_HFEKd (°)	87.44 ± 8.63	0.97 (0.92–0.99)	3.0 (2.3–4.6)
ROM_HFEKnd (°)	79.56 ± 8.63	0.95 (0.90–0.98)	2.9 (1.9–3.8)
ROM_HAFKd (°)	80.06 ± 7.73	0.97 (0.92–0.99)	2.2 (1.6–3.3)
ROM_HAFKnd (°)	74.44 ± 7.15	0.97 (0.93–0.99)	3.0 (2.3–4.6)
ROM_AFEKd (°)	38.09 ± 5.53	0.96 (0.90–0.98)	2.0 (1.5–3.1)
ROM_AFEKnd (°)	35.78 ± 5.11	0.94 (0.89–0.97)	3.3 (2.5–4.8)
ROM_AFFKd (°)	38.56 ± 5.93	0.92 (0.87–0.97)	2.7 (1.9–3.5)
ROM_AFFKnd (°)	36.25 ± 5.46	0.88 (0.69–0.91)	5.1 (4.0–8.1)
MMd (kg)	6.40 ± 0.86	-	-
MMnd (kg)	6.24 ± 0.81	-	-

ICC: intraclass correlation coefficient; CV: coefficient of variation; CI: confidence interval; °: grades; D: dominant; ND: non-dominant; 505-COD: 505 change of direction test; CMJ: countermovement jump; SBJ: standing broad jump; LAT: lateral jump; ROM: range of motion; HFEK: hip flexion with extended knee; HAFK: hip abduction with flexed knee; AFEK: ankle flexion with extended knee; AFFK: ankle flexion with flexed knee; MM: muscle mass.

**Table 2:** Mean interlimb asymmetry values and derived injury risk.

Fitness Test	Mean Asymmetry ± SD (%)	Injury Risk
505-COD	3.58 ± 3.54 ^$^	No ^&^
CMJ	14.50 ± 9.80 *^,$^	Yes ^&^
SBJ	4.24 ± 4.16 *	No ^&^
LAT	5.65 ± 3.09 *	No ^&^
ROM_HFEK	8.80 ± 7.66 *	Yes ^#^
ROM_HAFK	6.89 ± 5.13 *	No ^#^
ROM_AFEK	5.96 ± 4.05 *	No ^#^
ROM_AFFK	5.56 ± 8.04 *	No ^#^
MM	2.34 ± 1.64	No ^&^

505-COD: 505 change of direction test; CMJ: countermovement jump; SBJ: standing broad jump; LAT: lateral jump; ROM: range of motion; HFEK: hip flexion with extended knee; HAFK: hip abduction with flexed knee; AFEK: ankle flexion with extended knee; AFFK: ankle flexion with flexed knee; MM: muscle mass. * Higher interlimb asymmetry scores in comparison to 505-COD test (*p* < 0.05). ^$^ Higher interlimb asymmetry scores in comparison to MM test (*p* < 0.05). ^&^ Injury risk stablished in the threshold of 10% relating to Rohman et al. (2015) [21]. ^#^ Injury risk stablished in the threshold of 8% relating to López-Valenciano et al. (2019) [22].

**Table 3:** Pearson r correlations (95% confidence intervals) between the different interlimb asymmetry scores.

Fitness Test	505-COD	CMJ	SBJ	LAT	ROM_HFEK	ROM_HAFK	ROM_AFEK	ROM_AFFK
505-COD	-	-	-	-	-	-	-	-
CMJ	0.16 (−0.36–0.61)	-	-	-	-	-	-	-
SBJ	−0.13 (−0.59–0.39)	−0.07 (−0.50–0.49)	-	-	-	-	-	-
LAT	0.27 (−0.26–0.67)	−0.32 (−0.70–0.21)	−0.08 (−0.55–0.44)	-	-	-	-	-
ROM_HFEK	0.06 (−0.45–0.54)	0.11 (−0.41–0.58)	0.39 (−0.13–0.74)	0.09 (−0.42–0.56)	-	-	-	-
ROM_HAFK	−0.03 (−0.52–0.47)	−0.15 (−0.60–0.38)	0.47 (−0.30–0.78)	0.03 (−0.47–0.52)	0.42 (−0.10-0.76)	-	-	-
ROM_AFEK	0.32 (−0.21–0.70)	−0.27 (−0.68–0.26)	0.02 (−0.48–0.51)	0.18 (−0.35–0.62)	0.01 (−0.49–0.50)	0.17 (−0.36–0.61)	-	-
ROM_AFFK	−0.19 (−0.63–0.33)	−0.33 (−0.71–0.20)	0.44 (−0.07–0.77)	−0.07 (−0.54–0.45)	-0.49 (−0.59–0.39)	−0.16 (−0.61–0.36)	−0.28 (−0.68–0.26)	-
MM	−0.22 (−0.64–0.31)	−0.04 (−0.53–0.46)	−0.43 (−0.76–0.09)	0.01 (−0.49–0.50)	−0.31 (−0.70–0.22)	−0.21 (−0.64–0.32)	−0.39 (−0.74-0.13)	0.12 (−0.40–0.58)

505-COD: 505 change of direction test; CMJ: countermovement jump; SBJ: standing broad jump; LAT: lateral jump; ROM: range of motion; HFEK: hip flexion with extended knee; HAFK: hip abduction with flexed knee; AFEK: ankle flexion with extended knee; AFFK: ankle flexion with flexed knee; MM: muscle mass.

**Table 4:** Mean ROM values and derived injury risk.

Fitness Test	Mean ± SD	Reference Value	Injury Risk
ROM_HFEKd (°)	87.44 ± 8.63	<70° ^1^	No
ROM_HFEKnd (°)	79.56 ± 8.63		No
ROM_HAFKd (°)	80.06 ± 7.73	<50° ^2^	No
ROM_HAFKnd (°)	74.44 ± 7.15		No
ROM_AFEKd (°)	38.09 ± 5.53	<30° ^3^	No
ROM_AFEKnd (°)	35.78 ± 5.11		No
ROM_AFFKd (°)	38.56 ± 5.93	<40° ^4^	Yes
ROM_AFFKnd (°)	36.25 ± 5.46		Yes

°: grades; D: dominant; ND: non-dominant; ROM: range of motion; HFEK: hip flexion with extended knee; HAFK: hip abduction with flexed knee; AFEK: ankle flexion with extended knee; AFFK: ankle flexion with flexed knee. ^1^ Reference value for injury risk relating to Palmer and Epler (2002) [23]. ^2^ Reference value for injury risk relating to Gerhardt (1994) [24]. ^3^ Reference value for injury risk relating to Norris (2004) [25]. ^4^ Reference value for injury risk relating to Norris (2004) [25].

## Data Availability

The data presented in this study are available on request from the corresponding author.
